# Advanced Glycation End Products Promote PGE_2_ Production in Ca9-22 Cells via RAGE/TLR4-Mediated PKC–NF-κB Pathway

**DOI:** 10.3390/cells14231911

**Published:** 2025-12-02

**Authors:** Misae Ono, Natsuko Tanabe, Risa Ichikawa, Keiko Tomita, Soichiro Manaka, Hideaki Seki, Yuri Imai, Mayu Aoki, Yuma Masai, Tadahiro Takayama, Naoto Suzuki, Shuichi Sato

**Affiliations:** 1 Division of Applied Oral Sciences, Nihon University Graduate School of Dentistry, 1-8-13 Kanda-Surugadai, Chiyoda-ku, Tokyo 101-8310, Japan; 2 Department of Periodontology, Nihon University School of Dentistry, 1-8-13 Kanda-Surugadai, Chiyoda-ku, Tokyo 101-8310, Japan; 3 Division of Functional Morphology, Dental Research Center, Nihon University School of Dentistry, 1-8-13 Kanda-Surugadai, Chiyoda-ku, Tokyo 101-8310, Japan; 4 Department of Biochemistry, Nihon University School of Dentistry, 1-8-13 Kanda-Surugadai, Chiyoda-ku, Tokyo 101-8310, Japan; 5 Department of Anesthesiology, Nihon University School of Dentistry, 1-8-13 Kanda-Surugadai, Chiyoda-ku, Tokyo 101-8310, Japan; 6 Department of Orthodontics, Nihon University School of Dentistry, 1-8-13 Kanda-Surugadai, Chiyoda-ku, Tokyo 101-8310, Japan

**Keywords:** AGEs, COX2, TLR4 NF-κB, PGE_2_

## Abstract

Advanced glycation end products (AGEs) are compounds that accumulate in hyperglycemic states, contributing significantly to the development of diabetes and its complications, including the exacerbation of periodontal disease. We hypothesized that AGEs affect the expression of inflammatory mediators in gingival cells, thus contributing to the increased severity of periodontitis observed in diabetic patients. Thus, we stimulated the gingival epithelial carcinoma-derived cell line, Ca9-22, with AGEs and examined their effect on the expression of prostaglandin E_2_ (PGE_2_) and its primary synthesizing enzyme, cyclooxygenase 2 (COX2), key inflammatory mediators in periodontitis. AGEs significantly increased the expression levels of COX2 (n = 6, *p* < 0.001) and the production of PGE_2_ (n = 5, *p* < 0.05) compared to untreated control and bovine serum albumin (BSA) groups. The receptor for AGEs (RAGE) inhibitor FPS-ZM1 blocked the AGEs-stimulatory effects on COX2 (n = 7, *p* < 0.01), PGE_2_ (n = 6, *p* < 0.001), and Toll-like receptor 4 (TLR4) expression (n = 7, *p* < 0.001). Furthermore, AGEs induced the phosphorylation of protein kinase C (p-PKC) via the TLR4 pathway (n = 7, *p* < 0.01). Crucially, AGEs enhanced NF-κB nuclear accumulation, which was inhibited by blocking either RAGE (n = 5, *p* < 0.0001) or TLR4 (n = 5, *p* < 0.0001). In conclusion, these findings demonstrate that AGEs increase PGE_2_ production in Ca9-22 cells primarily through a signaling cascade involving RAGE and the TLR4-PKC-NF-κB pathway. Our results suggest TLR4 as a critical mediator that contributes to AGEs-induced inflammation.

## 1. Introduction

Advanced glycation end products (AGEs) are a chemically diverse group of compounds, formed both endogenously and exogenously [1]. They are created through a non-enzymatic modification process where the carbonyl groups of reducing sugars bind to free amine groups on molecules [1]. While AGEs form in moderate amounts under normal physiological conditions, their production is greatly accelerated by the high availability of glucose in continuous hyperglycemia. As an earlier in vivo study demonstrated, AGEs levels in rhesus monkeys’ serum positively correlated with their fasting blood glucose levels, mirroring the progression of diabetes. The initial responses are reversible, depending on the concentration of glucose in the serum. AGEs accumulate in every body fluid and are considered a primary cause of diabetic complications [2].

Diabetes mellitus (DM) is a metabolic disorder resulting from impaired insulin secretion, defective insulin action, or a combination of both. It is also an inflammatory condition, marked by a persistent low level of inflammation and an increase in circulating pro-inflammatory cytokines and acute-phase proteins. Although inflammation is typically a beneficial process aimed at restoring tissue balance, it can cause tissue damage when its magnitude or duration becomes excessive. Type 2 diabetes (T2DM) exacerbates periodontitis by intensifying inflammation within osteoblasts, osteocytes, gingival fibroblasts, periodontal ligament cells, and gingival epithelial cells, all of which are involved in periodontal tissue. Periodontal disease is an inflammatory oral condition caused by Gram-negative bacteria that leads to the destruction of periodontal tissues. The connection between T2DM and periodontitis is bidirectional, as suggested by many prior reports [3,4,5].

Periodontitis is initiated by lipopolysaccharide (LPS), an endotoxin derived from Gram-negative bacteria that infects periodontal tissue, resulting in inflammation and tissue destruction. LPS promotes the synthesis of inflammatory mediators, such as prostaglandin E_2_ (PGE_2_), which is produced by cyclooxygenase 2 (COX2) [6,7,8,9]. Both COX2 and PGE_2_ play a significant role in the development of inflammation. COX2 is an enzyme that converts arachidonic acid into eicosanoid lipid mediators like PGE_2_, which is not stored in cells but is produced in response to specific stimuli [10,11,12,13]. These mediators then promote both the gingival epithelial cells and the alveolar bones, causing periodontitis. However, the implication of AGEs on induced inflammatory mediators, such as PGE_2_, and the downstream mechanism of AGEs-induced inflammation in the gingival cells are elusive.

The receptor for advanced glycation end products (RAGE) is an N-glycosylated 35 kDa membrane-bound protein and a member of the immunoglobulin superfamily. It serves a key role in the innate immune response by recognizing various patterns and acting as a receptor for several ligands, including AGEs, S100 proteins, LPS, β-amyloids, and C3a-anaphylatoxin [14]. AGEs also increase the expression of Toll-like receptor 4 (TLR4), a receptor for LPS that activates nuclear factor-kappa B (NF-κB), leading to inflammation and the secretion of inflammatory mediators in various cells [7,15,16]. Furthermore, RAGE silencing inhibited the expression of TLR4, which is the receptor for both AGEs and LPS, as well as the phosphorylation of NF-κB in human umbilical vein endothelial cells and human retinal endothelial cells [17]. These reports suggest that AGEs-RAGE might affect TLR4-induced inflammation, such as periodontitis.

Protein kinase C (PKC), a family of serine/threonine kinases, governs key cellular signals necessary for activation, proliferation, differentiation, and survival [18]. The 12 or more existing PKC isoforms are grouped into three types: Classical PKC (α, β, γ) relies on diacylglycerol (DAG) and calcium; Novel PKC (δ, ε) is DAG-dependent but calcium-independent; and Atypical PKC (ζ, λ) is independent of both DAG and calcium [19]. PKC mediated NF-κB activation in various cells in some previous studies [18,19,20,21]. However, the effects of AGEs on the implication of TLR4 expression and NF-κB activation including PKC, which are involved in inflammation in periodontal tissue, particularly in gingival epithelial cells, are elusive. Hence, we hypothesized that AGEs affect the expression of inflammatory mediators through the RAGE/TLR4-PKC pathway, which involves the activation of NF-κB, and may contribute to the severity of periodontal disease in diabetic patients.

## 2. Materials and Methods

### 2.1. Cell Culture

The Ca9-22 human gingival carcinoma cell line (Riken BioResource Center, Tsukuba, Japan) was used as the gingival epithelial cell model. Cells were maintained in α-Minimal Essential Medium (FUJIFILMWako Pure Chemical, Osaka, Japan) supplemented with 10% (*v*/*v*) heat-inactivated fetal bovine serum (HyClone Laboratories, Logan, UT, USA) and 1% (*v*/*v*) Penicillin–Streptomycin–Amphotericin B suspension (FUJIFILMWako Pure Chemical) at 37 °C in a humidified atmosphere of 95% air and 5% CO_2_. Cells were either treated with 100 μg/mL AGEs or left untreated.

### 2.2. Preparation of AGEs

AGEs were prepared by incubating 50 mg/mL bovine serum albumin (BSA; FUJIFILMWako Pure Chemical) and 0.1 M DL-glyceraldehyde (Sigma-Aldrich, St. Louis, MO, USA) under sterile conditions in 0.2 M phosphate-buffered saline (PBS; pH 7.4) containing 5 mM diethylenetriamine pentametric acid (Nacalai Tesque, Kyoto, Japan) at 37 °C for 7 days. Then, low-molecular-weight reactants and aldehydes were removed using a PD-10 column (GE Healthcare Bio-Sciences AB, Uppsala, Sweden) and dialyzed against PBS [13,22,23,24,25]. AGEs were aliquoted to 1 mg/mL using Protein Assay Dye Reagent Concentrate (Bio-Rad Laboratories, Hercules, CA, USA), then stored at −20 °C before being diluted in cell culture medium to a final concentration of 100 µg/mL and used in the experiments [13].

### 2.3. Real-Time Polymerase Chain Reaction (Real-Time PCR)

Total RNA was isolated after 72 h of culture using the RNeasy Mini Kit (QIAGEN, Valencia, CA, USA). RNA concentration was measured using a NanoDrop 1000 spectrophotometer (Thermo Fisher Scientific, Wilmington, DE, USA). Complementary DNA (cDNA) was synthesized from 250 ng of DNase-treated total RNA using PrimeScript™ RT Master Mix (Takara Bio, Shiga, Japan), and real-time PCR was performed using TB Green^®^ Premix Ex Taq™ II (Takara Bio). Primers for COX2 were: forward 5′-CTGCGCCTTTTCAAGGATGG-3′ and reverse 5′-CCCCACAGCAAACCGTAGAT-3′. β-actin served as the internal reference, with the following primers: forward 5′-TGGCACCCAGCACAATGAA-3′ and reverse 5′-CTAAGTCATAGTCCGCCTAGAAGCA-3′. Each 25 μL PCR reaction contained 12.5 μL of TB Green^®^ Premix Ex Taq™ II, 0.5 μL (10 µM) of each primer, 9.5 μL of dH_2_O, and 2 μL (0.5 μg) of cDNA. Reactions were performed in a Thermal Cycler Dice Real-Time System II (Takara Bio), with 35 cycles at 95 °C for 5 s and 60 °C for 20 s. All reactions were performed in triplicate. Specificity of the amplified products was confirmed by melting curve analysis. COX2 mRNA levels were calculated using the ΔCt method.

### 2.4. Western Blotting

Cell lysates were prepared using the radioimmunoprecipitation assay lysis buffer (ATTO, Tokyo, Japan), containing protease inhibitor cocktail set III (EMD Millipore Corporation, CA, USA). Protein concentration was determined using an RC DC Protein Assay Kit (Bio-Rad Laboratories). First, 40 µg of protein were separated by sodium dodecyl sulfate-polyacrylamide gel electrophoresis and transferred to polyvinylidene difluoride membranes. Membranes were blocked with Block-Ace™ (KAC CO., Ltd., Hyogo, Japan) and incubated overnight at 4 °C with primary antibodies at 1:500 dilution in Can Get Signal Solution 1 (TOYOBO, Osaka, Japan). Primary antibodies included COX2 (cat#sc-376861, Santa Cruz Biotechnology, Dallas, TX, USA), TLR4 (cat#sc-293072, Santa Cruz Biotechnology), RAGE (cat#sc-365154, Santa Cruz Biotechnology), p-PKC (cat#9375) or PKC (cat#46809) (Cell Signaling Technology, MA, USA) or and β-actin (cat#sc-47778, Santa Cruz Biotechnology). Following primary antibody incubation, membranes were washed with Tris-buffered saline-Tween and incubated with HRP-conjugated Mouse IgGκ light chain binding protein (cat#sc-516102, Santa Cruz Biotechnology) or HRP-conjugated mouse anti-rabbit IgG (cat#2357, Santa Cruz Biotechnology) containing Can Get Signal Solution 2 (TOYOBO) at a 1:2500 dilution 1 h at room temperature as secondary antibody. β-actin served as the internal standard, and the protein bands were detected using Clarity Max™ Western ECL Substrate (Bio-Rad). Finally, immunoreactive proteins were visualized using an Amersham™ ImageQuant™ 800 system (Cytiva, Tokyo, Japan). We conducted five independent experiments: samples were collected from cell culture through stimulation, and the process was repeated five times. We also determined the protein expression using Western blotting. The target proteins, which were detected in Western blotting, and beta-actin were detected on the same membrane in a single experiment; the bands were quantified at the pixel using Fiji software (https://imagej.net/software/fiji/downloads, accessed on 5 April 2024) [26].

### 2.5. Enzyme-Linked Immune-Sorbent Assay (ELISA)

After 72 h of AGEs stimulation, the cells were cultured in serum-free medium for 24 h. The concentrations of PGE_2_ in the culture medium were determined using a commercially available ELISA kit (PGE_2_: R&D Systems, Minneapolis, MN, USA) according to the manufacturer’s instructions. PGE_2_ was corrected with the protein concentrations in the cell lysate, which were determined by the RC DC protein assay kit 1 (Bio-Rad Laboratories).

### 2.6. Immunofluorescence: The Nuclear Accumulation of NF-κB

Cells were seeded on glass coverslips, fixed with methanol for 30 min at −20 °C, and blocked with Block-Ace™ (KAC Co., Ltd.) for 1 h at room temperature. Next, they were incubated for 1 h each with rabbit monoclonal antibodies against NF-κB p65 (cat#8242, Cell Signaling Technology) and Alexa Fluor 488-conjugated goat anti-rabbit secondary antibody (Thermo Fisher Scientific) in blocking solution at room temperature. Nuclei were counterstained using ProLong™ Glass Antifade Mountant with NucBlue™ Stain (Thermo Fisher Scientific). Fluorescence signals were detected, and images were acquired using an All-in-One Fluorescence Microscope BZ-X810 (KEYENCE, Osaka, Japan). Negative control was stained in untreated control preparations when the primary anti-body was omitted (Supplemental Figure S1). For each sample, images were randomly captured at 5 points per coverslip to quantify NF-κB p65 nuclear accumulation. Cells were considered positive for nuclear accumulation of NF-κB p65 when its nuclear fluorescence intensity exceeded that of the cytoplasm. The percentage of cells positive for nuclear NF-κB p65 accumulation relative to total cells was calculated for each sample.

### 2.7. Statistical Analysis

Data represent means ± standard deviation from 4–6 independent experiments performed in triplicate. Data normality was assessed using the Shapiro–Wilk test or Kolmogorov–Smirnov test. Group differences were analyzed using one-way analysis of variance, followed by Tukey’s multiple comparison test, or Kruskal–Wallis test, followed by Dunn’s multiple comparison test. Differences were considered statistically significant at *p* < 0.05. All analyses were conducted using GraphPad Prism version 10.4.2 (GraphPad Software, San Diego, CA, USA).

## 3. Results

### 3.1. AGEs Increased COX2 and PGE_2_ Production in Ca9-22 Cells

**Figure 1:**
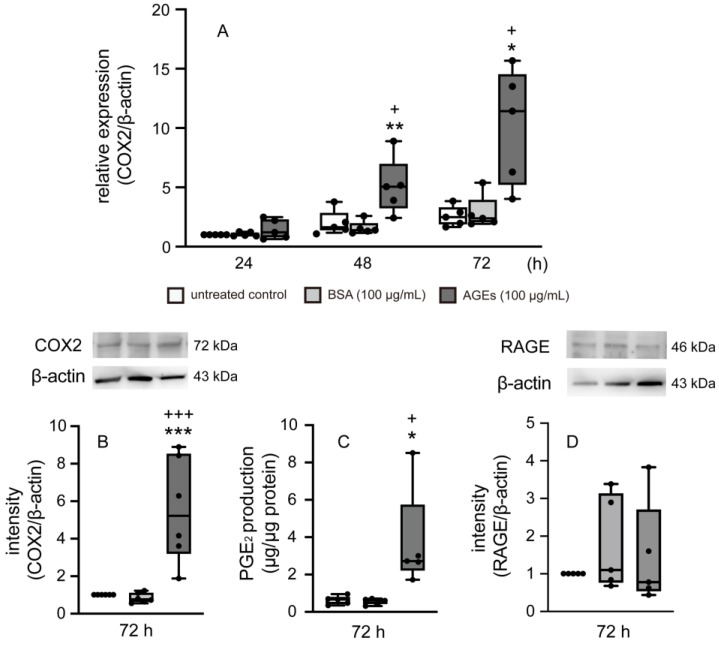
The mRNA expression of COX2 (**A**) in cells stimulated with BSA (100 µg/mL), AGEs (100 µg/mL), or left unstimulated (untreated control) was assessed for up to 72 h of culture using real-time PCR. The protein expression of COX2 (**B**) and RAGE (**D**) were assessed 72 h after AGEs stimulation using Western blotting. The production of PGE_2_ (**C**) on 72 h based on ELISA. The data in (**A**,**B**,**D**) represent the mean ± SD from five independent experiments conducted in triplicate. One-way ANOVA was employed for group comparisons, with Tukey’s post hoc test used for multiple comparisons among all groups. * *p* < 0.05, ** *p* < 0.01, *** *p* < 0.001 vs. untreated control; + *p* < 0.05, +++ *p* < 0.001 vs. BSA. The data of (**C**) represent the mean ± SD from six independent experiments conducted in triplicate. One-way ANOVA was employed for group comparisons, with Tukey’s post hoc test used for multiple comparisons among all groups. * *p* < 0.05, vs. untreated control; + *p* < 0.05 vs. BSA.

To examine the effect of AGEs on the synthesis of PGE_2_, we assessed Ca9-22 cells, which were found to express both COX2 and RAGE at the mRNA level (Figure 1A,B,D). COX2 gene expression was detected at 24, 48, and 72 h, irrespective of AGEs. AGEs significantly increased the mRNA and protein levels of COX2 in Ca9-22 cells by 48 h of culture compared to BSA, and AGEs also significantly increased COX2 mRNA levels by 72 h of culture compared to untreated control and BSA (Figure 1A). AGEs significantly increased protein levels of COX2 at 72 h of culture compared to untreated control and BSA (Figure 1B). Furthermore, AGEs also induced PGE_2_ production at 72 h relative to both the untreated control and BSA (Figure 1C). However, AGEs did not affect the mRNA expression of RAGE at 72 h compared to the control and BSA groups (Figure 1D).

### 3.2. RAGE Regulated the Expression of COX2, TLR4, and PGE_2_ Production in Ca9-22 Cells

**Figure 2:**
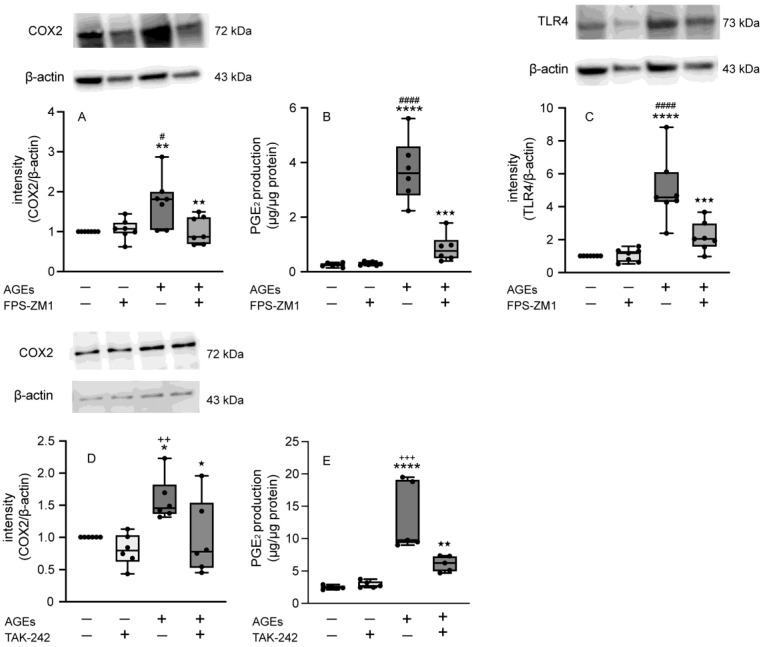
The protein expression of COX2 (**A**) and TLR4 (**C**) was stimulated with or without AGEs (100 µg/mL), FPS-ZM1 (20 µM), or left unstimulated (untreated control) and was assessed on 72 h of culture using western blotting. The production of PGE_2_ (**B**) was stimulated with or without AGEs (100 µg/mL), FPS-ZM1 (20 µM), or left unstimulated (untreated control) and was measured 72 h based on ELISA. The protein expression of COX2 (**D**) was stimulated with or without AGEs (100 µg/mL), TAK-242 (5 µM), or left unstimulated (untreated control) and was assessed at 72 h of culture using Western blotting. The production of PGE_2_ (**E**) was stimulated with or without AGEs (100 µg/mL), TAK-242 (5 µM), or left unstimulated (untreated control) and was measured 72 h based on ELISA. The data in A and C represent the mean ± SD from seven independent experiments conducted in triplicate. One-way ANOVA was employed for group comparisons, with Tukey’s post hoc test used for multiple comparisons among all groups. ** *p* < 0.001, **** *p* < 0.0001 vs. untreated control; # *p* < 0.01, #### *p* < 0.0001 vs. FPS-ZM1, ^★★^ *p* < 0.01, ^★★★^ *p* < 0.001 vs. AGEs. The data of B, D, and E represent the mean ± SD from six independent experiments conducted in triplicate. One-way ANOVA was employed for group comparisons, with Tukey’s post hoc test used for multiple comparisons among all groups. * *p* < 0.05, **** *p* < 0.0001 vs. untreated control; #### *p* < 0.01 vs. FPS-ZM1, ^++^ *p* < 0.01, ^+++^ *p* < 0.01 vs. TAK-242, ^★^ *p* < 0.05, ^★★★^ *p* < 0.001 vs. AGEs.

We then investigated the role of RAGE in the AGE-induced increase in COX2 and PGE_2_. The RAGE inhibitor FPS-ZM1 blocked the stimulatory effect of AGEs on both protein expression of COX2 and PGE_2_ production at 72 h (Figure 2A,B). In addition, AGEs increased the protein expression of TLR4, whereas FPS-ZM1 blocked the stimulatory effect of AGEs (Figure 2C). Next, we explored the effects of the TLR4 inhibitor TAK-242 (5 µM) on the protein expression of COX2 and PGE_2_ production in Ca9-22 cells. TAK-242 also blocked AGEs-induced COX2 protein expression and PGE_2_ production at 72 h. These results showed that RAGE and TLR4 were implicated in the AGE-induced expression of COX2 and PGE_2_ (Figure 2D,E).

### 3.3. AGEs Increased NF-κB Nuclear Accumulation in Ca9-22 Cells

**Figure 3:**
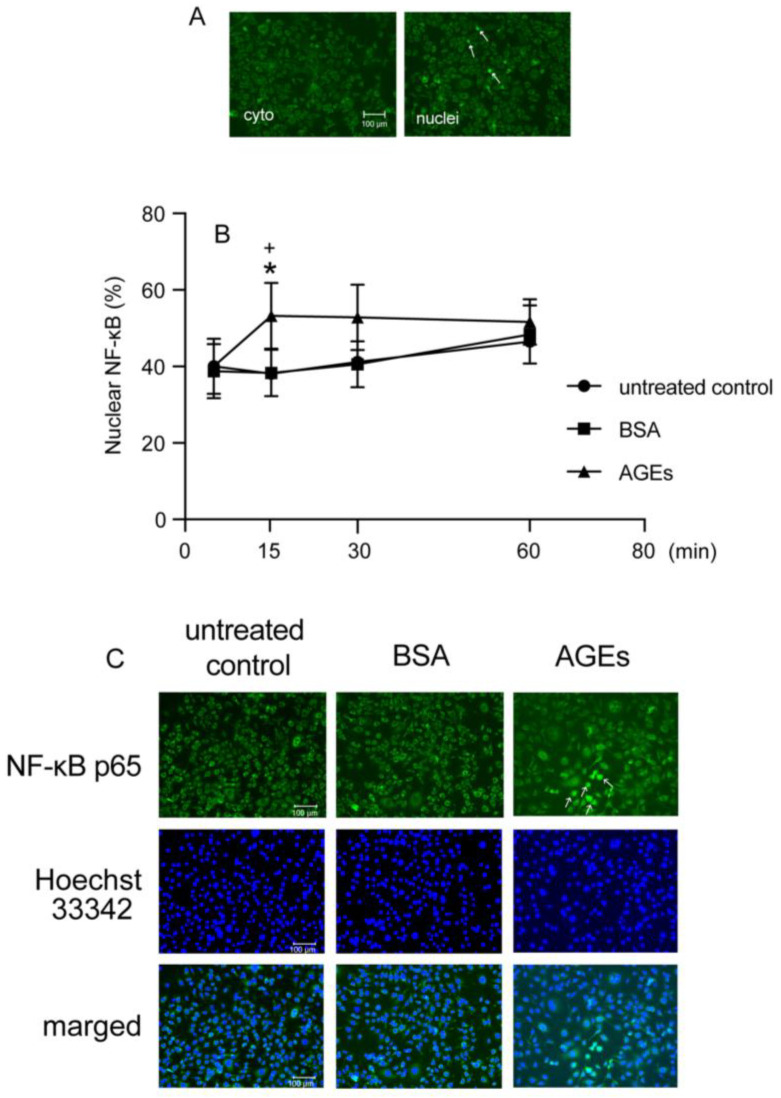
Cells plated on coverslips and stimulated with AGEs (100 μg/mL), treated with BSA (100 μg/mL), or left unstimulated (untreated control) for up to 60 min, and then were fixed with methanol. NF-κB p65 was assessed using immunofluorescence. Image of Ca9-22 cells cytoplasmic (cyto, (**A**)) NF-κB localization. Images of Ca9-22 cells showing NF-κB localized in the nuclei (nuclei, (**A**)). Box-and-whisker plot showing the percentage of cells with nuclear accumulation of NF-κB p65 (**B**). NF-κB localization was assessed using immunofluorescence. Image of AGEs-stimulated cells; arrows indicate that NF-κB localized in the nuclei (**C**). The data of B are expressed as the mean ± SD of five independent experiments. One-way ANOVA was employed for comparisons between groups, while Tukey’s post hoc test was employed for multiple comparisons among all groups. * *p* < 0.05 vs. untreated control; + *p* < 0.05 vs. BSA.

**Figure 4:**
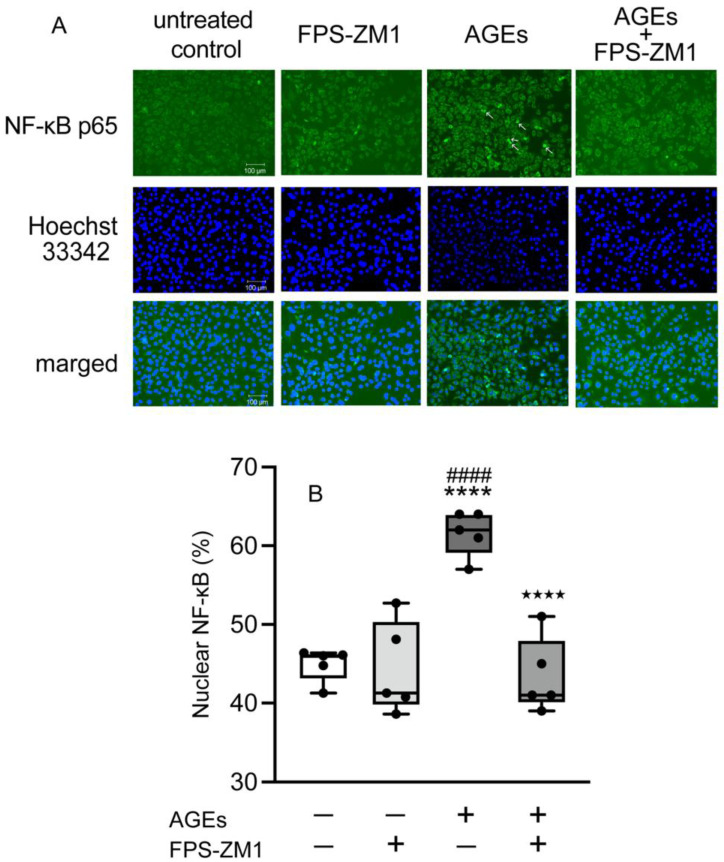
Cells plated on coverslips and stimulated with or without AGEs (100 μg/mL), treated with FPS-ZM1 (20 μM), or left unstimulated (untreated control) for 15 min, were fixed with methanol. NF-κB p65 was assessed using immunofluorescence. Image of AGEs-stimulated cells; arrows indicate that NF-κB localized in the nuclei (**A**). Box-and-whisker plot showing the percentage of cells with nuclear accumulation of NF-κB p65 (**B**). Data are expressed as the mean ± SD of five independent experiments. One-way ANOVA was employed for comparisons between groups, while Tukey’s post hoc test was employed for multiple comparisons among all groups. **** *p* < 0.0001 vs. untreated control; ^####^ *p* < 0.0001 vs. FPS-ZM1; ^★★★★^ *p* < 0.0001 vs. AGEs.

**Figure 5:**
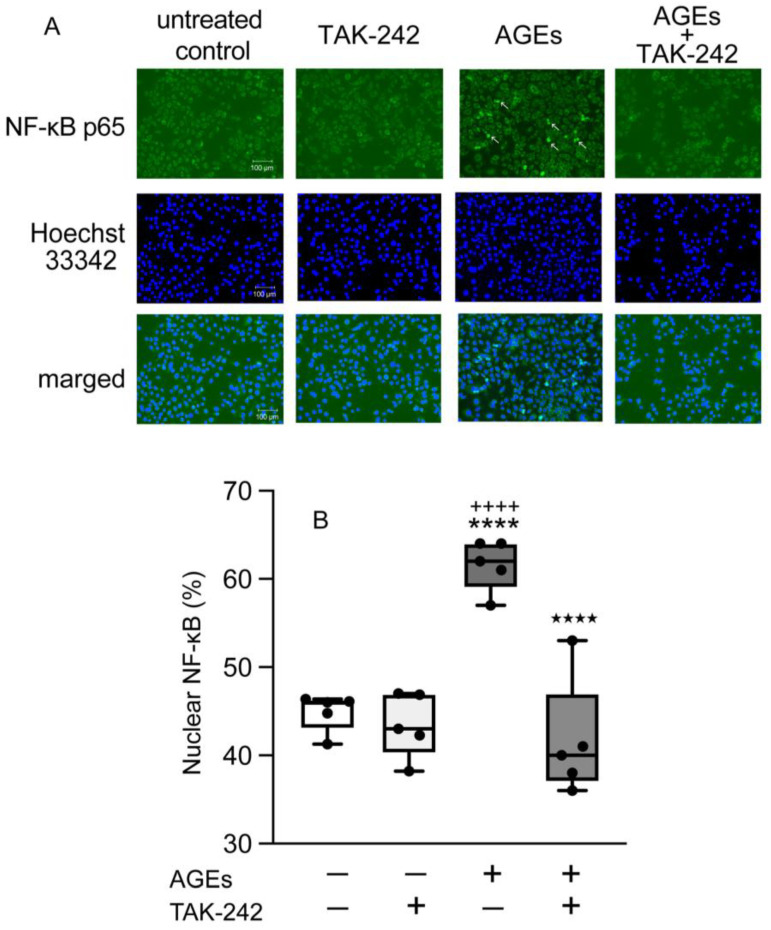
Cells plated on coverslips and stimulated with or without AGEs (100 μg/mL), treated with TAK-242 (5 μM), or left unstimulated (untreated control) for 15 min, were fixed with methanol. NF-κB p65 was assessed using immunofluorescence. Image of AGEs-stimulated cells; arrows indicate that NF-κB localized in the nuclei (**A**). Box-and-whisker plot showing the percentage of cells with nuclear accumulation of NF-κB p65 (**B**). Data are expressed as the mean ± SD of five independent experiments. One-way ANOVA was employed for comparisons between groups, while Tukey’s post hoc test was employed for multiple comparisons among all groups. **** *p* < 0.0001 vs. untreated control; ^++++^ *p* < 0.0001 vs. TAK-242; ^★★★★^ *p* < 0.0001 vs. AGEs.

To investigate the nuclear accumulation of NF-κB, we detected NF-κB by immunohistochemistry after AGEs stimulation. Figure 3A shows the accumulation of NF-κB cytosol (cyto) or nuclei (nuclei). AGEs induced the nuclear accumulation of NF-κB compared to untreated control and BSA at 15 min (Figure 3B,C). AGEs increased the NF-κB nuclear accumulation, whereas FPS-ZM1 blocked stimulatory effect of AGEs on 15 min (Figure 4A,B). TAK-242 also inhibited AGEs-induced NF-κB nuclear accumulation at 15 min (Figure 5A,B).

### 3.4. AGEs Increased p-PKC via TLR4 in Ca9-22 Cells

**Figure 6:**
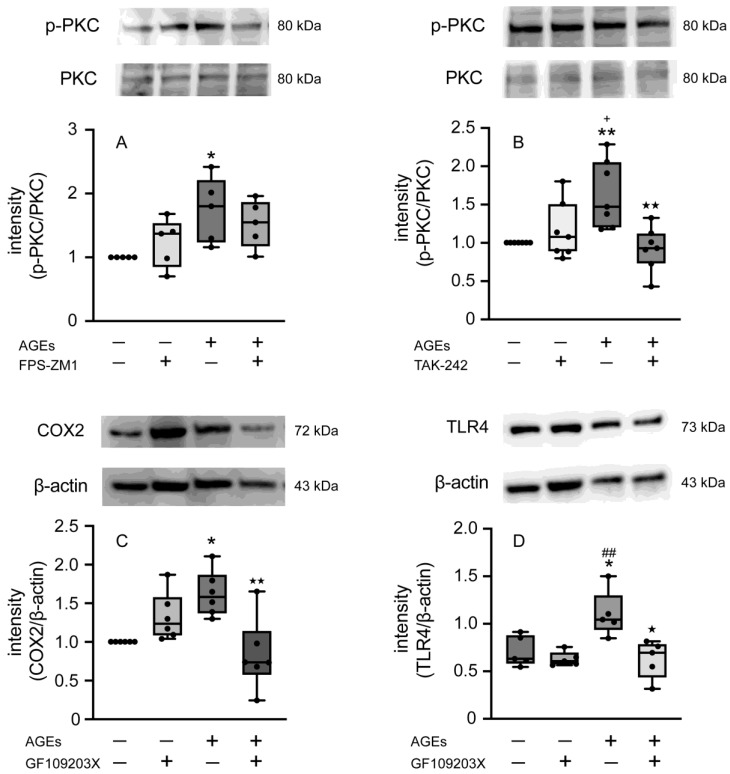
The protein expression of p-PKC (**A**) stimulated with AGEs (100 µg/mL), FPS-ZM1 (20 µM), or left unstimulated (untreated control) was assessed after 5 min using Western blotting. The protein expression of p-PKC (**B**) stimulated with AGEs (100 µg/mL), TAK-242 (5 µM) or left unstimulated (untreated control) was assessed on 5 min using western blotting. The protein expression of COX2 (**C**) and TLR4 (**D**) stimulated with AGEs (100 µg/mL), GF109203X (10 µM), or left unstimulated (untreated control) was assessed after 5 min using western blotting. The data of A and B represent the mean ± SD from five independent experiments conducted in triplicate. One-way ANOVA was employed for group comparisons, with Tukey’s post hoc test used for multiple comparisons among all groups. * *p* < 0.05, ** *p* < 0.01 vs. untreated control; ^+^ *p* < 0.05 vs. TAK-242, ^★^ *p* < 0.01 vs. AGEs. The data of C and D represent the mean ± SD from six independent experiments conducted in triplicate. One-way ANOVA was employed for group comparisons, with Tukey’s post hoc test used for multiple comparisons among all groups. * *p* < 0.05 vs. untreated control; ^##^ *p* < 0.01 vs. GF109230X, ^★^ *p* < 0.05, ^★★^ *p* < 0.01 vs. AGEs.

To investigate the signaling pathway of AGEs in Ca9-22 cells, we detected the protein expression of p-PKC by Western blotting treatment with or without FPS-ZM1, TAK-242, and stimulated AGEs. AGEs increased p-PKC at 5 min, and TAK-242 inhibited the stimulatory effect of AGEs; however, FPS-ZM1 did not affect p-PKC (Figure 6A,B). The PKC inhibitor GF109203X inhibited AGEs-induced COX2 and TLR4 protein levels (Figure 6C,D).

### 3.5. PKC-Mediated NF-κB Nuclear Accumulation in Ca9-22 Cells

**Figure 7:**
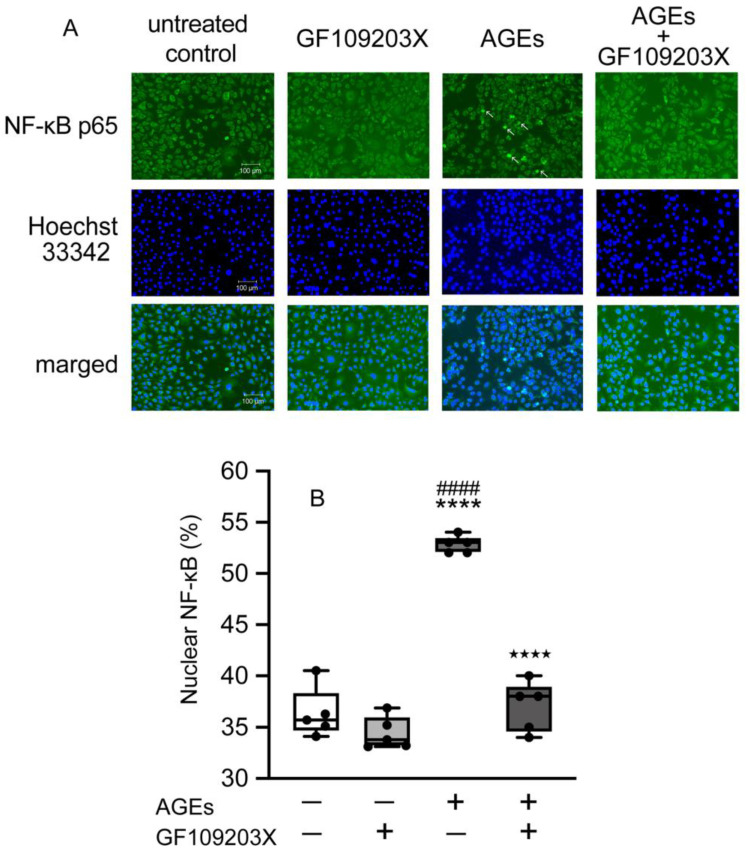
Cells plated on coverslips and stimulated with or without AGEs (100 μg/mL), treated with GF109203X (10 μM), or left unstimulated (untreated control) for 15 min, were fixed with methanol. NF-κB p65 was assessed using immunofluorescence. Image of AGEs-stimulated cells; arrows indicate that NF-κB localized in the nuclei (**A**). Box-and-whisker plot showing the percentage of cells with nuclear accumulation of NF-κB p65 (**B**). Data are expressed as the mean ± SD of five independent experiments. One-way ANOVA was employed for comparisons between groups, while Tukey’s post hoc test was employed for multiple comparisons among all groups. **** *p* < 0.0001 vs. untreated control; ^####^ *p* < 0.0001 vs. TAK-242; ^★★★★^ *p* < 0.0001 vs. AGEs.

To investigate the nuclear accumulation of NF-κB, we detected the NF-κB by immunohistochemistry after AGEs stimulation. AGEs increased the NF-κB nuclear accumulation, whereas GF109203X blocked stimulatory effect of AGEs on 15 min (Figure 7A,B).

## 4. Discussion

Periodontitis is primarily caused by poor oral hygiene, as well as a combination of genetic and environmental factors. It progresses from the initial stage, gingivitis, to periodontitis due to several factors, including a shift in dental plaque from aerobic, Gram-positive bacteria to anaerobic, Gram-negative bacteria, genetic changes, and alterations in the host environment [27,28]. While microbial agents are the direct cause, they also drive damaging inflammatory reactions in susceptible hosts via the formation of strongly adhesive biofilms [28,29].

DM, characterized by hyperglycemia, is a major metabolic disorder that alters the oral microbiota, leading to reduced bacterial diversity, with proteobacteria and firmicutes becoming dominant species [30]. The periodontium of diabetic individuals exhibits heightened inflammation, specifically an increase in NF-κB activation and the expression of cytokines (TNF and IL-1), all of which are triggered by high local concentrations of glucose, ROS, and AGEs [30]. Since blocking inflammatory mediators like IL-17, RANKL, and IL-6 only partially reverses these microbial changes in diabetic mice, the findings suggest a strong connection between inflammation, oral bacterial composition, and DM [28,30].

The systematic review of Thomas et al. reported that patients with chronic diabetic periodontal disease had elevated AGE levels compared to the non-diabetic group. Furthermore, the levels of both AGEs and RAGE were found to be associated with increased periodontal pocket depth (PPD) and clinical attachment loss (CAL). This review conclude that AGE and RAGE levels and expression differ significantly between diabetic and non-diabetic periodontitis patients, suggesting that these differences may influence the course and severity of periodontal disease [31]. *Tannerella forsythia*, implicated in periodontitis, produces methylglyoxal (MGO), a dicarbonyl compound. The levels of MGO correlate with the severity of periodontitis. MGO can induce inflammation either directly or through the formation of glycation products known as AGEs [32]. Kashket et al. reported that the common periodontal pathogen *Tannerella forsythia* and *Porphyromonas gingivalis* endogenously produce AGEs, which occur in gingival and periodontal tissues. In this regard, it is suggested that the MGO secreted by T. forsythia contributes to periodontal tissue destruction by impairing the healing function of gingival fibroblasts and causing endothelial cell dysfunction [33,34]. These previous reports suggest that periodontitis, which involves inflammation of periodontal tissue, is induced by AGEs, which are produced by host or microbial agents. AGEs significantly increased the expression of LCN2 in both human oral epithelial cells TR146 cells and Ca9-22 cells. Furthermore, AGEs induced LCN2 through the NF-κB pathway [35]. TNF-α and IL-1β were significantly induced by AGEs in mouse tubular epithelial cells [36]. These reports demonstrates that inflammatory responses have similar effects on tumor cells and primary cells originating from the same source. Thus, we investigated the effects of AGEs on the expression of inflammatory mediators, such as PGE_2_, and the mechanisms that elucidate inflammation induced by AGEs in gingival epithelial cells, specifically Ca9-22 cells, in the present study.

COX2 is one of the two isoforms of COX, a rate-limiting enzyme that converts arachidonic acid to prostaglandin. Unlike COX1, which is constitutively expressed and maintains homeostatic functions, COX2 is an inducible enzyme triggered by inflammatory and mitogenic stimuli [15,37,38] In this present study, AGEs significantly increased the mRNA and protein expression of COX2, and the production of PGE_2_, synthesized by COX2 (Figure 1). We next determined the effect of RAGE on the expression of COX2 and PGE_2_ induced by AGEs using RAGE inhibitor FPS-ZM1. AGEs increased the protein expression of COX2 and PGE_2_ production via RAGE (Figure 2A,B).

Toll-like receptors (TLRs), such as TLR2 and TLR4, are activated by bacterial components, and a signaling cascade begins. This cascade culminates in the nuclear accumulation of NF-κB, thereby enhancing the transcription of genes responsible for producing cytokines, chemokines, adhesion molecules, and other inflammatory factors associated with bacterial infection [21]. In addition, TLR4 is induced by DM conditions in many cell types, not only in response to bacterial infections, such as LPS stimulation [39,40]. Previous studies have shown that AGEs activate RAGE/TLR4-induced M1 macrophage polarization, which is dependent on vascular smooth muscle cell phenotypic conversion [16,41]. AGEs modified low density lipoprotein also induced inflammatory cytokine IL-6 through the activation of TLR4 in renal tubular epithelial cells [42]. These findings indicate that TLR4 was activated by AGEs. Then, we determined the protein expression of TLR4 treated with or without AGEs. In the present study, AGEs induced the protein expression of TLR4 (Figure 2C). Moreover, the TLR4 inhibitor TAK-242 inhibited COX-2 protein expression and PGE_2_ production (Figure 2D,E). Our results suggest that AGEs induced inflammation through the activation both RAGE and TLR4.

The transcriptional regulatory factor NF-κB plays a crucial role in controlling the expression of numerous immunoregulatory mediators essential for acute inflammation [43]. NF-κB is a family of transcription factors (chief heterodimer: p50 and p65 in the canonical pathway) that interacts with COX2 to regulate its transcription, as indicated by studies where inhibiting NF-κB p50 reduced COX2 expression. Under normal conditions, NF-κB remains inactive in the cytoplasm because its nuclear accumulation sequence is masked by the inhibitory protein IκB-α [44]. Inflammatory stimuli, including bacterial products like LPS and peptidoglycan, or pro-inflammatory cytokines, trigger the activation of the IKK complex (containing IKKα and IKKβ kinases) [45]. IKKs phosphorylate IκB-α at serines 32 and 36, marking it for subsequent ubiquitinylation and degradation by the 26S proteasome. Once IκB-α is degraded, NF-κB is free to move into the nucleus and activate target genes. Furthermore, studies have demonstrated that PKC, specifically the classical family, is involved in regulating this process by activating IKKs and promoting the necessary degradation of IκB-α [18,19,20,46,47]. AGEs increased the expression of TLR4 and enhanced the activation of NF-κB in breast cancer cell line MDA-MB-231 cells [48]. AGEs-induced inflammation affects TLR4 expression through RAGE in human umbilical vein endothelial cells and human retinal endothelial cells [17]. Thus, in the present study, we next determined the effect of NF-κB activation on AGEs stimulation in Ca9-22 cells. AGEs increased the nuclear accumulation of NF-κB at 15 min (Figure 3A), an effect blocked by both the RAGE inhibitor FPS-ZM1 and the TLR4 inhibitor TAK-242 (Figure 4 and Figure 5). These results clearly suggest that the NF-κB pathway is crucial for AGEs-induced COX2-PGE_2_ expression in Ca9-22 cells, requiring the engagement of both RAGE and TLR4. Asehnoune et al. reported that PKCα/β is essential for NF-κB accumulation via TLR4 by activating the IKK complex [21]. Some previous studies demonstrated that AGEs activate NF-κB through PKC in various epithelial cells [49,50]. In this regard, we focus on the implications of PKC-NF-κB, including RAGE and TLR4, on AGEs-induced PGE_2_ production in the present study. The role of PKC in this cascade was then investigated. Consistent with a role for TLR4, AGEs induced p-PKC at 5 min, and this stimulatory effect was entirely inhibited by the TLR4 inhibitor TAK-242 (Figure 6B). However, the RAGE inhibitor FPS-ZM1 did not affect p-PKC induction (Figure 6A). This finding suggests that while RAGE is indispensable for NF-κB nuclear accumulation and subsequent PGE_2_ production, it may not be the direct upstream activator of PKC phosphorylation. RAGE recognizes AGEs and is known to activate NF-κB via PKC-independent mechanisms, potentially involving p38 MAPK or ERK, which are established downstream effectors of RAGE in various cell types [51,52]. Our combined data suggest that this RAGE-mediated NF-κB activity then promotes the transcriptional upregulation of TLR4 (Figure 2C), creating a positive inflammatory feedback loop. The increased TLR4 then becomes the dominant receptor responsible for PKC phosphorylation upon continuous stimulation with AGEs. The increased TLR4 then becomes the dominant receptor responsible for the PKC phosphorylation upon continuous AGEs stimulation. The increased TLR4 then becomes the dominant receptor for the PKC phosphorylation upon continuous AGEs stimulation. Furthermore, the PKC inhibitor GF109203X inhibited both AGEs-induced COX2 and TLR4 protein expression (Figure 6C,D), suggesting that PKC acts as an important downstream molecule mediating the effects of activated TLR4. These results suggest a bipartite mechanism in which RAGE controls the expression level of TLR4, and TLR4, in turn, dictates the activation of the PKC-NF-κB cascade, thereby amplifying the overall inflammatory signal in gingival epithelial cells.

While a previous study identified a RAGE-TLR4-pPKCβ1 tripartite complex on dendritic cell membranes [53]. our results suggest that in Ca9-22 cells, RAGE’s primary role is regulatory, enabling the TLR4 signal. To elucidate further details of the RAGE-TLR4-PKC signaling pathway activated by advanced glycation end products (AGEs), it is necessary to investigate whether complex formation occurs in gingival epithelial cells in the future. Our findings indicate that AGEs may exacerbate inflammation in gingival epithelial cells not only by activating RAGE, but also by increasing the expression of TLR4, which is activated by both AGEs and LPS. These findings in this study demonstrate for the first time the downstream effect of AGEs-induced inflammation, which increases PGE_2_ and activates NF-κB through not only RAGE but also TLR4. AGEs may exacerbate inflammation in periodontitis by activating both RAGE and TLR4, clarifying the molecular link between diabetes and severe periodontal destruction. However, our study observed the basal mechanism of AGEs in promoting PGE_2_, one of the inflammatory mediators, in the human gingival carcinoma cell line Ca9-22, which exhibits an epithelial-like character in vitro. We would elucidate the effect of AGEs on exacerbating inflammation in periodontal tissue, including gingival epithelial cells, in future studies.

## 5. Conclusions

AGEs were found to increase the production of PGE_2_ via the RAGE/TLR4-NF-κB pathway in Ca9-22 cells. TLR4 plays a role in the increase in inflammatory mediators induced by AGEs. These findings indicate that AGEs may exacerbate LPS-induced inflammation via not only RAGE but also TLR4 in periodontitis.

## Data Availability

The data that support the findings of this study are available from the corresponding author upon reasonable request.

## Supplementary Materials

The following supporting information can be downloaded at: https://www.mdpi.com/article/10.3390/cells14231911/s1, Figure S1: The images of negative control on immunofluorescence.

## Abbreviations

The following abbreviations are used in this manuscript:

AGEs	advanced glycation end-products
DM	Diabetes mellitus
T2DM	type 2 diabetes
RAGE	receptor for advanced glycation end-products
IL-1	interleukin-1
IL-6	interleukin-6
IL-8	interleukin-8
IL-17	interleukin-17
TNFα	tumor necrosis factor-α
RANKL	receptor activator of nuclear factor-kappa B ligand
LPS	lipopolysaccharide
PGE_2_	prostaglandin E_2_
COX1	cyclooxygenase 1
COX2	cyclooxygenase 2
ELISA	enzyme-linked immuno-sorbent assay
PKC	protein kinase C
p-PKC	phosphorylated protein kinase C
NF-κB	nuclear factor-kappa B
TLR	Toll-like receptor
