# Supplementary material for: Advanced Glycation End Products Promote PGE2 Production in Ca9-22 Cells via RAGE/TLR4-Mediated PKC–NF-κB Pathway
Source: Cells. 2025 Dec 2;14(23):1911. doi: 10.3390/cells14231911 (PMC12691458; doi:10.3390/cells14231911)
Supplement: Supplementary file 1 [file cells-14-01911-s001.zip › cells-3947193-supplementary.pdf]

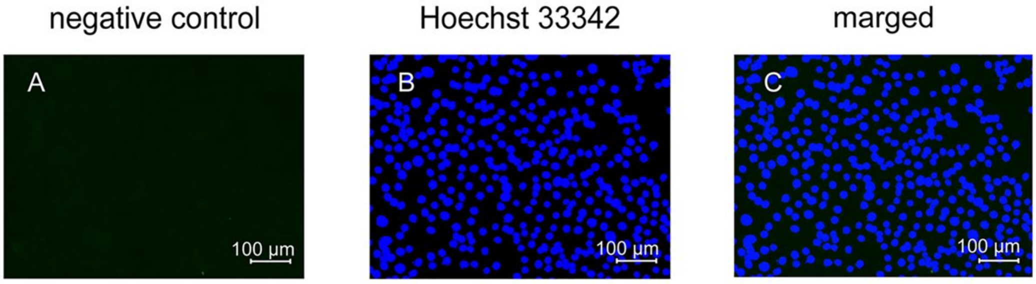

Supplemental Figure S1:The images of negative control on immunofluorescence. Negligible staining was observed in untreated control preparations when the primary antibody was omitted (A), and nuclei in the same cells were revealed by Hoechst 33342 staining (B). The image of C merged with A and B (C).
